# A Retrospective Review of Outcomes of Dental Treatment Performed for Special Needs Patients under General Anaesthesia: 2-Year Follow-Up

**DOI:** 10.1155/2014/748353

**Published:** 2014-12-24

**Authors:** Sreekanth Kumar Mallineni, Cynthia Kar Yung Yiu

**Affiliations:** ^1^Vasan Dental Care, Banjara Hills, Hyderabad, Telangana 500004, India; ^2^Abhiram Institute of Medical Sciences, Atmakur, Nellore, Andhra Pradesh 524322, India; ^3^Paediatric Dentistry and Orthodontics, Faculty of Dentistry, University of Hong Kong, Hong Kong

## Abstract

*Objective*. The purpose of the study was to evaluate the follow-up pattern of special needs patients (SNP) treated under general anaesthesia (GA) and the failure rates of different treatment procedures and restorative materials. *Study Design*. The treatment records of the patients who received dental treatment under GA during 2005 to 2009 were reviewed. The duration of follow-up periods, the outcomes of different treatment procedures, and the quality of different restorative materials were recorded and evaluated. Statistics were used for the comparison (SPSS 20.0). Pearson's chi-square test and post hoc analysis were used to evaluate the attendance of postoperative appointments and the associations of failure rates of different treatment procedures and restorative materials. Cohen kappa statistics was used for intraexaminer reliability. *Results*. A total of 177 patients were included in the study. The attendance of postoperative appointments showed a gradual decrease from 96% to 36% within 24 months (*P* < 0.05). Restorative procedures had the highest failure rates among all treatment procedures (*P* < 0.05). Stainless steel crowns showed higher survival rates among different restorative materials (*P* < 0.05). Pulp treatment in primary molars has higher success rate than primary incisors and canines. Composite restorations placed in primary canines have low survival rates. The intraexaminer reliability was good (*k* = 0.94). *Conclusion*. The attendance for postoperative follow-up appointments declined within two years. Restorative treatment was less successful when compared to preventive and pulp treatments. Stainless steel crowns were more reliable restorations with higher survival rates and composite restorations were less durable.

## 1. Introduction

Most of the studies on the use of general anaesthesia (GA) for dental treatment agreed on its appropriateness for facilitation of treatment for fearful and/or behaviourally resistant individuals. Many studies have reported that high anxiety levels and low level of cooperation and/or medical conditions are the possible reasons for the dental treatment under GA [1–4]. It has been reported that dental treatment should be carried out in a hospital-based setting for patients with special needs, who need comprehensive restorative and/or surgical procedures [5–7]. Furthermore, the decision should be made based on the age, level of cooperation, and dental and medical histories of the patients [4, 6, 8]. The implementation of the postoperative follow-ups allows the behaviour of child to improve and eventually minimizes the need of repeat GA for dental care [9].

Comprehensive treatment is the most important goal for dental treatment under GA for SNP. This should always be implemented with a preventive program, behavioural remodelling, and a follow-up appointment to avoid repeat GA [10]. Under GA, paediatric dentists may not follow the same treatment protocol as used in the dental clinic. To simplify treatment and reduce the risk of failure, tooth extraction is often a preferable choice than pulp therapies or root canal treatment in SNP when the pulp of a tooth is inflamed. Previous reports have described a wide range of restorative work provided to SNP under GA [4, 11]. Harrison and Roberts [2] reported that the underlying medical disorder may influence treatment planning under GA. Several studies have reported the clinical outcomes of dental treatment under GA for healthy patients [12–15]; however, very few studies have reported similar outcomes on SNP [5, 16]. Therefore, the purpose of the present study was to evaluate the follow-up pattern of the SNP that had received dental treatment under GA and to determine the failure rate of different treatment procedures and restorative materials.

## 2. Materials and Method

This is a retrospective study on the provision of dental treatment under GA for SNP from a major teaching dental hospital in Hong Kong. All these patients either were referred to the Paediatric Dentistry Clinic, Prince Philip Dental Hospital, by general dental practitioners or special needs school or directly attended the out-patient clinic with their parents or caregivers. The SNP received clinical examination and the appropriate radiographs were taken during the first screening appointment. The patients were placed on the waiting list for treatment under general anaesthesia at Queen Mary Hospital, Hong Kong SAR, if other behavioural management techniques have been attempted and failed.

Comprehensive dental treatment for patients with special needs under GA was carried out weekly at the Queen Mary Hospital. The patient was usually admitted to the hospital in the morning on the scheduled date of the surgery. On the morning of the surgery, following a detailed oral examination, details of the provisional treatment plan were explained to the parents. The parents were also informed that the treatment plan might need to be modified during the operation, depending on the circumstances. There was no facility for taking radiographs in the operation theatre. In general, the operative procedures were routinely performed under rubber dam isolation, while tooth extractions were performed after the completion of the restorative treatment. Local anaesthetics were used for tooth extractions. All the patients were discharged on the same day following the operation, if there were no complications from GA. However, if the patients were found to be present with any complications, they would be kept under observation until the vital signs were stable. The follow-up appointment was scheduled two weeks postoperatively at Paediatric Dentistry Clinic, Prince Philip Dental Hospital, after treatment under GA.

All the cases treated under GA with complete records were included in the present study (*N* = 177). The cases with incomplete records, cleft lip, and/or palate and treated by other disciplines were excluded from the study. Data were obtained from the clinical records of patients who received dental treatment under GA during the time period of January 2005 to December 2009. Details of patient folder number, gender, date of birth, date of treatment, and treatment procedures, including tooth type and restoration type, were collected and cautiously recorded. The prescription of pre- and postoperative radiographs was recorded. Data from five follow-up appointments, 2 weeks, 6 months, 12 months, 18 months, and 24 months, were collected. At these follow-up appointments, clinical and radiographic examinations were performed to evaluate the failures of treatment provided under GA.

In addition to the follow-up appointments, the patients might attend an emergency visit as unscheduled recall at the Paediatric Dentistry Clinic, Prince Philip Dental Hospital. Some of the patients who missed their scheduled appointments could attend as unscheduled emergency appointment. The findings from these appointments were also included in the final evaluation. The criteria established for the evaluation of success and failure for treatments procedures (preventive procedure, restoration procedure, pulp therapies, and root canal treatments) were shown as follows.

*Established Criteria for Evaluation of Failures in Different Treatment Procedures and Restorations*

1. preventive procedures:
  - dislodgement,
  - incomplete coverage with open margins,
  - secondary caries;
2. restorations:
  - dislodgement,
  - secondary caries,
  - poorly adapted,
  - complete loss,
  - apical radiolucency,
  - pain,
  - loosen crowns (stainless steel crowns),
  - tooth extracted/ mobility due to pathology;
3. pulp therapy:
  - swelling,
  - abscess,
  - pain,
  - apical radiolucency,
  - perforation,
  - tooth extracted/mobility due to pathology.

For the purpose of comparison, the treatment procedures were classified as preventive (fissure sealants), restorative (amalgam, composites resin, glass ionomer cements, and stainless steel crowns), pulp therapy (pulpotomy and pulpectomy), and root canal treatment. The treatment procedures and materials used for restoration were evaluated based on the tooth type. The reason for this was to find out the tooth, which commonly required retreatment after treatment under GA. The computer printouts, which contain all the necessary details for the study, were screened carefully. Following a washout period of 2 weeks, ten percent of the patient records were randomly selected to evaluate the intraexaminer reliability.

Data was analyzed using the SPSS (Version 20.0, Chicago, Illinois) software. Pearson's chi-square test and post hoc analysis were used for the comparison of failure rates of different procedures and restorative materials by the end of 24 months, while Cohen kappa statistics was used for intraexaminer reliability.

## 3. Results

**Figure 1:**
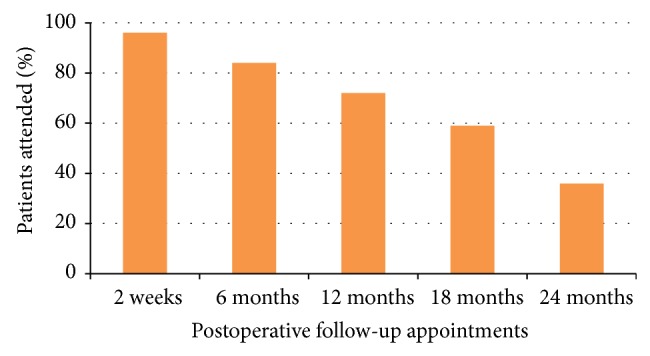
Patients' attendance based on follow-up appointments.

One hundred and ten (62%) males and 67 (38%) females were involved in the study. The mean age of the patients at the time they received GA was 12.3 ± 10.5 years, with a range from 1.8 years to 50 years. Over 96% of the patients attended the first postoperative appointment 2 weeks after treatment, while only 64 (36%) patients completed the 24-month follow-up. The patients' attendance of follow-up appointments declined significantly from 96% in the first follow-up appointment to 36% in the 24-month follow-up appointment (Figure 1) (*P* < 0.05). 31 (18%) of the patients failed to have the postoperative radiographs taken during follow-up appointments.

A total of 1,978 treatment procedures were performed in the SNP under GA during 2005 and 2011, among these 343 (17%) were preventive procedures, 985 (50%) were restorative, 152 (7.6%) were pulp therapy, 490 (25%) were extractions, and 8 (0.4%) were root canal treatments. The mean and standard deviations for the different treatment procedures (preventive, restorative, pulp therapy, extractions, and root canal treatments) were 1.9 ± 2.2, 5.6 ± 3.9, 0.85 ± 1.4, 2.8 ± 3.2, and 0.05 ± 0.2, respectively.

The failure rates of different treatment procedures at the end of 24-month follow-up are shown in Table 1. Statistically significant associations existed between failure rates and treatment procedures (*P* < 0.01). The failure rate of restorative procedures was the highest among the three treatment procedures. The failure rate of restorative procedures was significantly higher than pulpotomy/pulpectomy (*P* < 0.001). No failures were found in teeth that received root canal treatment.

The failures rates according to the type of restorative material are shown in Table 2. Statistically significant associations existed between failure rates and restorative materials (*P* < 0.01). The stainless steel crowns had the lowest failure rate (3.8%), which was followed by glass ionomer cements (10.2%), amalgams (13.0%), and composite restorations (22.7%). The failure rate of stainless steel crowns was significantly higher than composite restorations (*P* < 0.001) and amalgam restorations (*P* < 0.05). Thus, stainless steel crowns were the best restorative material among all four materials.

The failure rates for each primary and permanent tooth type according to the four different treatment procedures are shown in Table 3. Significant association existed between failure rates of restorative procedures and the primary tooth type (*P* < 0.05). The results showed that 40.3% of the restorative procedures placed in primary canines needed replacement, which was significantly higher than 21.6% in the primary incisors and 10.3% in the primary molars (*P* < 0.01). Conversely, significant association existed between failure rates of pulp treatment and the primary tooth type (*P* < 0.001). Pulp treatment in primary incisors (51.7%) had a significantly higher failure rate when compared to primary molars (6.4%) and primary canines (33.3%) (*P* < 0.01). Significant association existed between failure rate of preventive procedure and the permanent tooth type. The failure rate of preventive procedures was significantly higher for permanent molars (17.9%), when compared to permanent premolars (2.1%) (*P* < 0.05). No significant association was found between the failure rate of restorative procedures and different permanent tooth type.

The failure rates for each primary and permanent tooth type according to the four restorative materials are shown in Table 4. Significant association existed between the failure rate of composite restorations and primary tooth type (*P* < 0.001). Forty-five percent of the composite restorations placed in primary canines required retreatment. The failure rate of composite restorations in primary canines was significantly higher than primary molars (*P* < 0.001). No significant association was found between the failure rate of glass ionomer cements and primary tooth type. No significant association was found between the failure rate of restorative materials and permanent tooth type. The Cohen kappa statistics showed good intraexaminer reliability agreement (*k* = 0.94).

## 4. Discussion

The patients included in the present study had significant medical histories and developmental disabilities. Low follow-up rates were evident after dental treatment under GA. The follow-up attendance of the patients declined from 96% to 36% from the immediate postoperative follow-up appointment (2 weeks) to the follow-up at 24 months. These findings were in agreement with previous studies [5, 12–14]; however, the follow-up rates were different from other studies [5, 12–15]. The reason for this was the time frames for the follow-up were not similar. It has been reported that only patients who had pain or noticed any failure of restoration were more likely to visit dental clinic after treatment under GA [17]. Berkowitz and coworkers [5] evaluated the clinical outcomes of patients with nursing caries 4–6 months postoperatively following treatment under GA. They found that parents are unresponsive to follow-up care with 71.4% of the patients failing to attend the review appointments. In a retrospective study of 96 anxious or handicapped patients who received dental treatment under GA, 88% of the patients attended the follow-up appointment held 2 months postoperatively [16]. Foster and coworkers [18] reported that more than 50% patients failed the first two-week postoperative appointment. These findings are in contrast to our study, where 96% of the SNP attended the first follow-up appointment. Special needs patients have either significant medical histories or developmental disabilities. Majority of them have limited ability to perform daily oral hygiene measures. Follow-up appointments are, therefore, important to them as they are at high caries risk. The purpose for immediate follow-up appointment was postoperative evaluation, reinforcement of oral hygiene, and dietary counselling to reduce the occurrence of new carious lesions. Topical fluoride varnish application was performed for all the patients who attended the follow-up appointment. However, the lower return rate of SNP for follow-up after dental rehabilitation may affect the success rate of these preventive measures.

Restorative procedures had higher failure rate when compared to preventive procedures, pulp therapies, and root canal treatments. Pulp treatments had the lowest failure rate among all restorative procedures. It has been reported that the failure rates for pulpotomies were less than 2% [12], 2.9% [14], and 16.3% [15]. Pulp treatments in primary incisors had higher failure rate than canines and molars and almost 50% of pulp therapy performed in primary incisors required further treatment. Procedures with lower success rate should be avoided to achieve the best possible clinical outcomes for treatment under GA. Therefore, extraction should be considered for severely broken down primary incisors.

Amalgam, composites, and glass ionomer restorations had lower survival rates, when compared to stainless steel crowns. Over 20% of teeth restored with composite restorations required further treatment where the findings were similar to prior studies [14, 15]. The failure rate of amalgam restorations in our study was lower than previous studies [12–15]. However, the utilization of amalgam was very low (4.7%) in our study when compared to other studies. In our study, stainless steel crowns were more successful than other restorative materials with only 3.8% failure rate. Stainless steel crowns are the most reliable restorations and a more cost-effective restorative material for primary molars, while composite restorations are the least durable for SNP treated under GA.

The failure rate of restorative procedures was high in primary canines. Composite restorations have higher failure rate in primary canines than incisors and molars. These high failure rates may be due to the difficulties encountered in restoring badly broken down primary canines. In view of the poor treatment outcome, extraction may be a better option for the badly broken down primary canines in SNP. For permanent teeth, more attention should be given to improve the retention of the composite restorations during cavity preparation, bonding, and placement of restorations.

It is undoubtedly in the interest of the SNP to provide comfortable, definitive, durable, and functional restorations with the least amount of time spent under GA. A proper treatment plan, taking into account the patient's underlying medical conditions, the outcomes of the various treatment procedures, and restorative materials, is necessary to ensure the provision of high-quality dental service to SNP and to avoid the need for repeat dental general anaesthesia [9, 18].

The kappa statistics performed for intraexaminer reliability showed excellent agreement (*k* = 0.94). The limitation for this study was that 31 patients (17%) did not have pre- and postoperative radiographs as they were unmanageable for radiographs. For these patients the failure rates were evaluated based on clinical records retrospectively. Radiographs were prescribed for these SNP based on the clinical evaluation.

## 5. Conclusions

Based on our study, we concluded that the attendance for postoperative follow-up appointments declined from 96% to 36% within two years. The restorative procedures showed higher failures rates than preventive procedures and pulp treatments. Stainless steel crowns are more reliable restorations and composite restorations are less durable. Composite restorations placed in primary canines showed higher failure rates. Pulp treatment in primary molars has higher success rate than primary incisors and canines. Proper formulation and execution of treatment plan are essential to ensure a more positive outcome for special needs patient treated under general anaesthesia. Implementation of a postoperative review programme, which provides the opportunity to implement preventive care to SNP, modify their behaviours, and motivate the parents/caregivers in prevention of dental disease, is vital to reduce the risk of repeat dental anaesthesia.

## Figures and Tables

**Table 1:** Failures rates of different treatment procedures.

Treatment procedures	Total number of procedures	Number of failed procedures	Failure rate (%)	Post hoc
Preventive procedures	343	41	11.9	
Restorative procedures	985	160	16.2	b
Pulpotomy/pulpectomy	152	14	9.2	
Root canal treatment	8	—	—^*^	

^*^Not included in statistical analysis.

Statistically significant at *P* < 0.01, Pearson's chi-square test.

Post hoc *P* < 0.001, a = preventive procedures versus restorative procedures; b = restorative procedures versus pulpotomy/pulpectomy; c = preventive procedures versus pulpotomy/pulpectomy.

**Table 2:** Failures rates of different restorative procedures.

Restorative materials	Total number of restorations	Number of failed restorations	Failure rate (%)	Post hoc
Amalgam restorations	46	6	13.0	c
Composite restorations	611	139	22.7	
Glass ionomer cement	39	4	10.2	
Stainless steel crowns	289	11	3.8	e

Statistically significant at *P* < 0.01; Pearson's chi-square test.

Post hoc *P* < 0.05, a = amalgam versus composite; b = amalgam versus GIC; c = amalgam versus SSC; d = composite versus GIC.

e = composite versus SSC; f = GIC versus SSC.

**Table 3:** Failure rates of different treatment procedures by tooth type.

Treatment procedures	Failure rate (%)				Overall *P* value	Post hoc
	Primary tooth					
	Incisors	Canines		Molars		

Preventive procedures	—	—		13.3	—	
Restorative procedures	21.6	40.3		10.3	*P* < 0.01	a, b, c
Pulpotomy/pulpectomy	51.7	33.3		6.4	*P* < 0.01	a, b, c
Root canal treatment	—	—		—	—	

	Permanent tooth					
	Incisors	Canines	Premolars	Molars		

Preventive procedures	0	—	2.1	17.9	*P* < 0.05	F
Restorative procedures	19.6	26	3.1	14.2	*P* > 0.05	
Pulpotomy/pulpectomy	0	—	—	—	—	
Root canal treatment	0	—	—	—		

Post hoc *P* < 0.01, a = primary incisor versus primary canine; b = primary incisor versus primary molar; c = primary canine versus primary molar.

Post hoc *P* < 0.01, A = permanent incisors versus permanent canines; B = permanent incisors versus premolars; C = permanent incisors versus permanent molars; D = permanent canines versus premolars; E = permanent canines versus permanent molars; F = premolars versus permanent molars.

**Table 4:** Failure rates of different restorative materials by tooth type.

Restorative materials	Failure rate (%)				Overall *P* value	Post hoc
	Primary tooth					
	Incisors	Canines		Molars		

Amalgam	—	—		—	—	
Composite	22	45		19	*P* < 0.001	c
Glass ionomer cement	12.5	25		20	*P* > 0.05	
Stainless steel crowns	—	—		3.9	—	

	Permanent tooth					
	Incisors	Canines	Premolars	Molars		

Amalgam	—	—	10	13.8	*P* > 0.05	—
Composite	20.6	23.5	0	15.5	*P* > 0.05	—
Glass ionomer cement	0	33.3	0	0	*P* > 0.05	—
Stainless steel crowns	—	—	—	0	—	—

Post hoc *P* < 0.001, a = primary incisor versus primary canine; b = primary incisor versus primary molar; c = primary canine versus primary molar.
